# Art driven by visual representations of chemical space

**DOI:** 10.1186/s13321-023-00770-4

**Published:** 2023-10-21

**Authors:** Daniela Gaytán-Hernández, Ana L. Chávez-Hernández, Edgar López-López, Jazmín Miranda-Salas, Fernanda I. Saldívar-González, José L. Medina-Franco

**Affiliations:** 1 DIFACQUIM Research Group, Department of Pharmacy, School of Chemistry, Universidad Nacional Autónoma de México, Avenida Universidad 3000, 04510 Mexico City, Mexico; 2 Department of Chemistry and Graduate Program in Pharmacology, Center for Research and Advanced Studies of the National Polytechnic Institute, 07000 Mexico City, Mexico

**Keywords:** Artwork, Chemical space, Chemical multiverse, Chemoinformatics, Data visualization, Education, Food chemistry, Foodinformatics, Molecular representation, Open science

## Abstract

Science and art have been connected for centuries. With the development of new computational methods, new scientific disciplines have emerged, such as computational chemistry, and related fields, such as cheminformatics. Chemoinformatics is grounded on the chemical space concept: a multi-descriptor space in which chemical structures are described. In several practical applications, visual representations of the chemical space of compound datasets are low-dimensional plots helpful in identifying patterns. However, the authors propose that the plots can also be used as artistic expressions. This manuscript introduces an approach to merging art with chemoinformatics through visual and artistic representations of chemical space. As case studies, we portray the chemical space of food chemicals and other compounds to generate visually appealing graphs with twofold benefits: sharing chemical knowledge and developing pieces of art driven by chemoinformatics. The art driven by chemical space visualization will help increase the application of chemistry and art and contribute to general education and dissemination of chemoinformatics and chemistry through artistic expressions. All the code and data sets to reproduce the visual representation of the chemical space presented in the manuscript are freely available at https://github.com/DIFACQUIM/Art-Driven-by-Visual-Representations-of-Chemical-Space-. *Scientific contribution*: Chemical space as a concept to create digital art and as a tool to train and introduce students to cheminformatics.

**Graphical Abstract:**

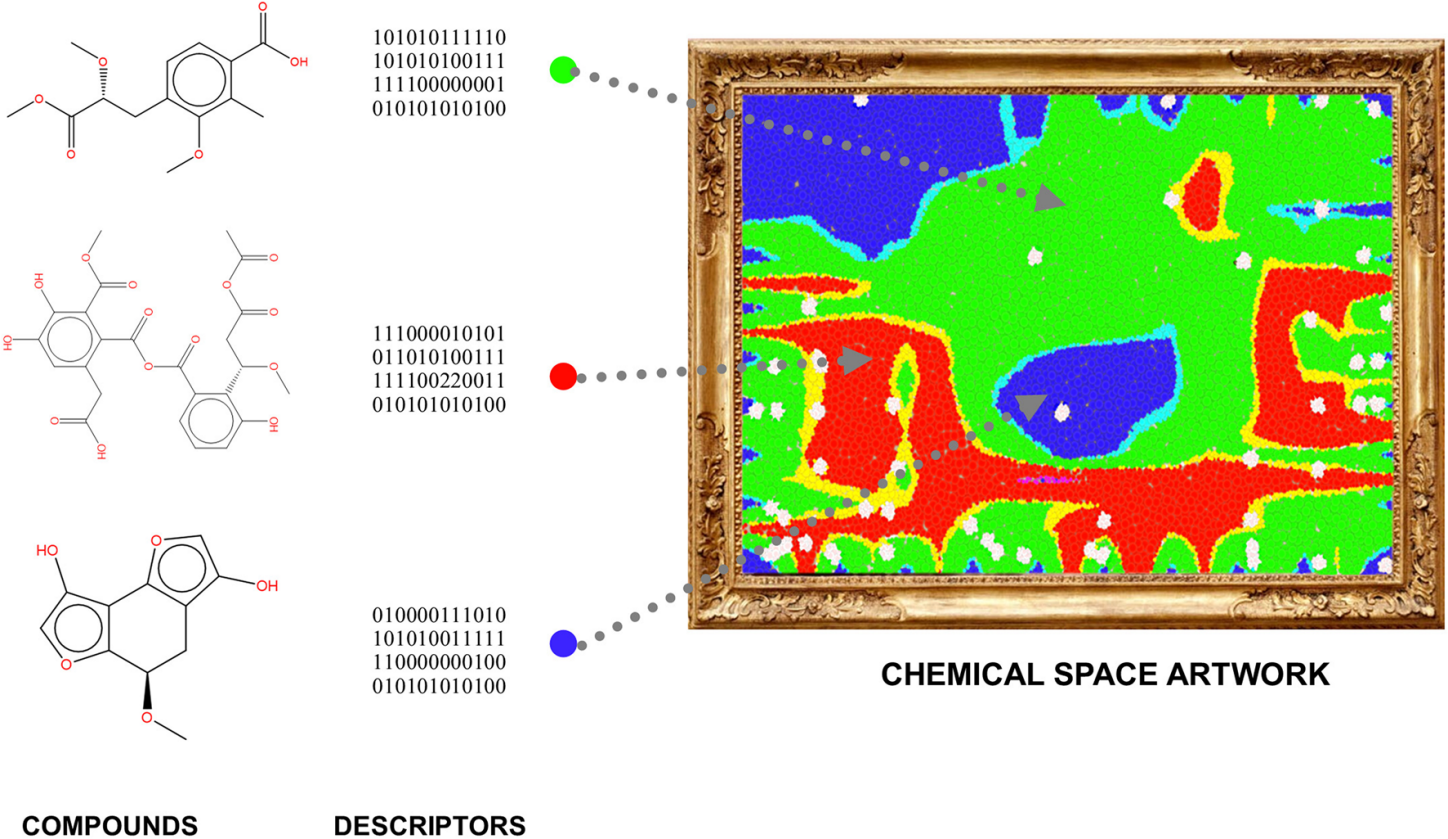

**Supplementary Information:**

The online version contains supplementary material available at 10.1186/s13321-023-00770-4.

## Introduction

Art can be considered as the set of activities and products of human beings with aesthetic, ethical, and communication objectives that impact individuals or societies [1]. Its impact may seek to transmit ideas, emotions, needs, concerns, or values [2]. Science can be considered an art tool that makes the materialization of ideas possible and delimits the ideas of artists. What is important about science is not only that it has served to enable the work to be executed. What is fundamental is that it has allowed it to be imagined. Furthermore, scientific knowledge allows for a more profound interpretation of art.

Historically, the relationship between science and art has existed since humans created art. One example is chemistry, a scientific discipline that historically has had a symbiotic relationship with art and has determined its respective evolutions. Among the many interactions of chemistry in art are the development of pigments and spectroscopic techniques, materials for conservation and restoration, to name just a few [3, 4].

**Fig. 1:**
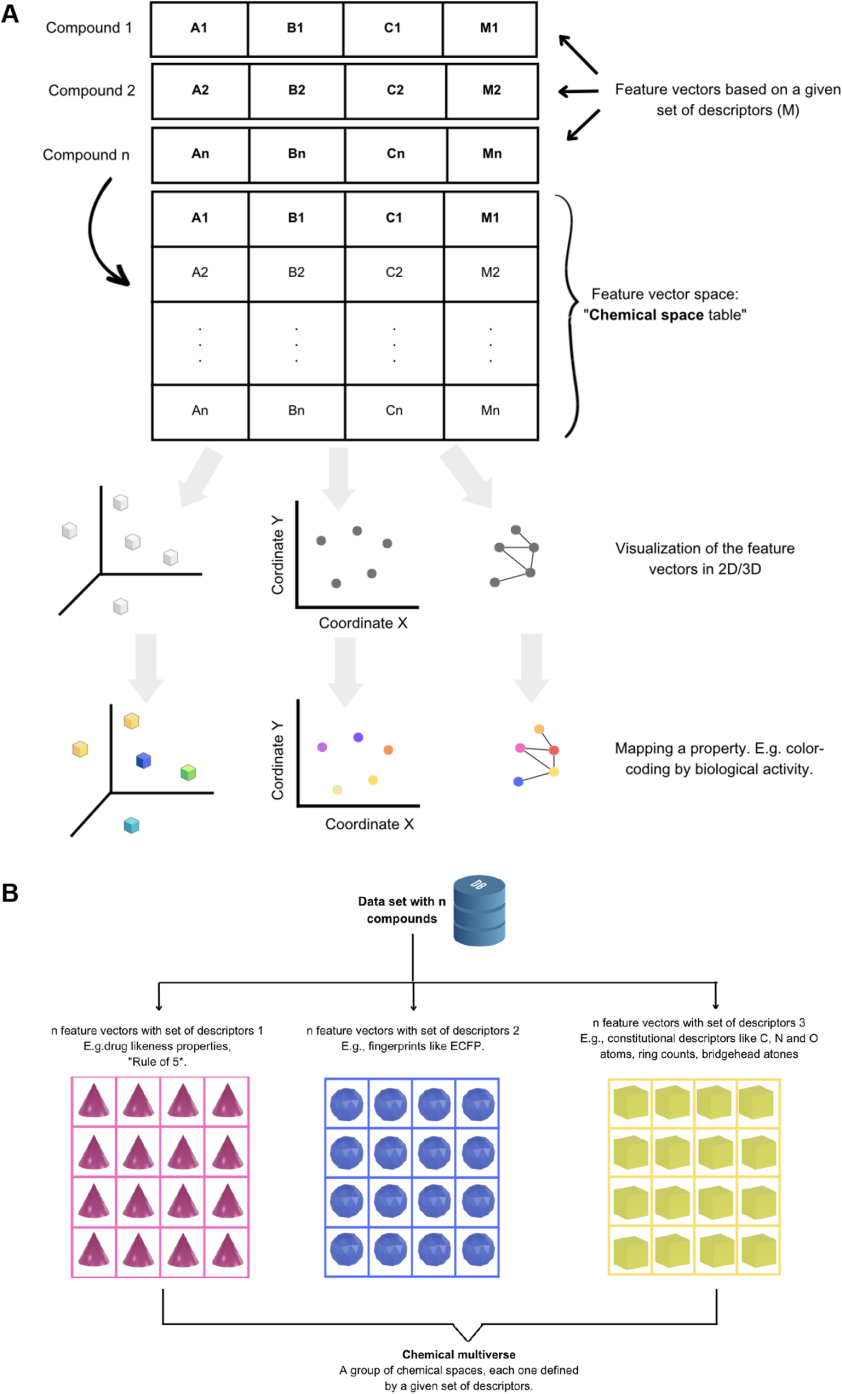
Schematic concept of **A** chemical space and its visual representation in low-dimensions. **B** Schematic representation of a chemical multiverse for a hypothetical data set of n compounds: descriptors of different design (continuous properties, molecular fingerprints, constitutional descriptors, etc.) can lead to alternative chemical spaces for the same data set

The advent of computers gave rise first to computational chemistry and then chemoinformatics. Chemoinformatics, also frequently referred to in the literature as cheminformatics [5] aims to manage and organize information, visualize chemical space, perform data mining, and establish mathematical relationships between chemical structures and properties. While bioinformatics focuses on biologically relevant macromolecules, chemoinformatics is focused on small compounds [6]. As an independent theoretical discipline, chemoinformatics relies on the chemical space concept [7–10]. Understanding the concept of chemical space within and outside chemoinformatics can be complicated. Generally, this concept has been accompanied by various images that seek to represent characteristics that chemists have assigned according to the inherent purposes of their research, leaving aside the aesthetic composition that, in turn, can contribute to deepening and communicating beyond the common sense, which associates thinking to an operation that excludes its connections with the affections, sensitivity, and creation. In Chemoinformatics, chemical space has been defined as a chemical descriptor vector space (cf. Fig. 1A) set by the numerical vector X encoding property or molecular structure aspects as elements of the descriptor vector X [11]. As such, chemoinformatics methods strongly depend on molecular representation and numerical descriptors [12]. There are many descriptors whose selection will depend on the type of molecules studied, for example, organic, inorganic, small molecules, peptides (whose size can differ significantly), natural products, and food chemicals, to name a few. For small molecules (e.g., molecular weight < 1000 Da), it is common to use as descriptors molecular fingerprints [13, 14], whole molecule properties (e.g., properties of pharmaceutical relevance [15, 16]), and sub-structures such as molecular scaffolds [17]. Figure 1A shows a schematic representation of the concept of chemical space, e.g., a chemical space table as a matrix where compounds are the rows and the numerical descriptors are the columns. Graphical and reduction dimension techniques are used to map the usually large multi-dimensional spaces into two or three dimensions that can be plotted and easily visualized.

Since the chemical space of a set of compounds is not unique and will depend on the set of descriptors chosen to describe it, multiple chemical spaces are theoretically possible for the same data set. Continuing this line of thinking, a chemical multiverse was proposed recently and defined as “the group of numerical vectors that describe differently the same set of molecules.” An alternative definition of the chemical multiverse is a “group of multiple chemical spaces, each defined by a given set of descriptors—a group of “descriptor universes” [7]. The chemical multiverse concept is represented in Fig. 1B.

Chemical spaces and chemical multiverses are, like many other types of analysis, frequently analyzed through data visualization techniques (Fig. 1). Indeed, data visualization is widely used in science and other areas to effectively summarize and communicate data to produce information and, ultimately, knowledge. Extensive reviews have been published concerning the visualization of chemical spaces [9, 10]. As reviewed, there are multiple methods of visualization, such as principal component analysis (PCA) [18], t-distributed stochastic neighbor embedding (t-SNE) [19], Tree MAP (TMAP) [20], self-organizing map (SOM) [21–23], and the generative topographic mapping (GTM) [24]. Each one will have advantages and disadvantages. As emphasized above, the visualization of a given data set will depend on the type of descriptors used.

The visual representation of chemical spaces can lead to visually appealing figures, particularly if appropriate color schemes are used. The visually attractive settings are used to emphasize patterns in the chemistry data to facilitate visual information extraction. For instance, to highlight grouping or clustering in the chemistry data or to rapidly identify patterns in the structure–property landscapes. At the same time, the visually attractive graphs can be for the chemistry expert and non-expert, a visually appealing graph, or a digital “painting” or work of art. In other words, the graph or digital painting is driven by chemical structures and descriptors. Therefore, the person generating the chemical space representation could be considered a chemical space artist who can communicate not only chemical data and information but even emotions if the chemical structures are associated with a personal, emotional, or another type of feeling the “artist” / author want to communicate through the visualization, e.g., an artistic expression.

In this sense, the concept of chemical space also opens up the possibility of searching for new representations that have to do with the need to configure another image of thought, and think in a novel fashion; it is a creative task and is similar to art.

This manuscript proposes the general notion of generating visual representations of chemical space and chemical multiverses as a means of chemical communication that produces new experiences and, in parallel, artistic expressions. To illustrate the proposal, we generated chemical space visualizations of four flavor categories from an extensive public database of food chemicals, FooDB [25], using different descriptors and molecular fingerprints. We considered four flavor categories, as detailed in the Methods section. The concept would further promote art driven by chemoinformatics and can be expanded to other information-related disciplines, such as bioinformatics. Using different descriptors and visualization methods, we show examples of chemical multiverse visualizations of four flavor categories from FooDB and other chemical compounds.

## Methods

### Data sets

**Fig. 2:**
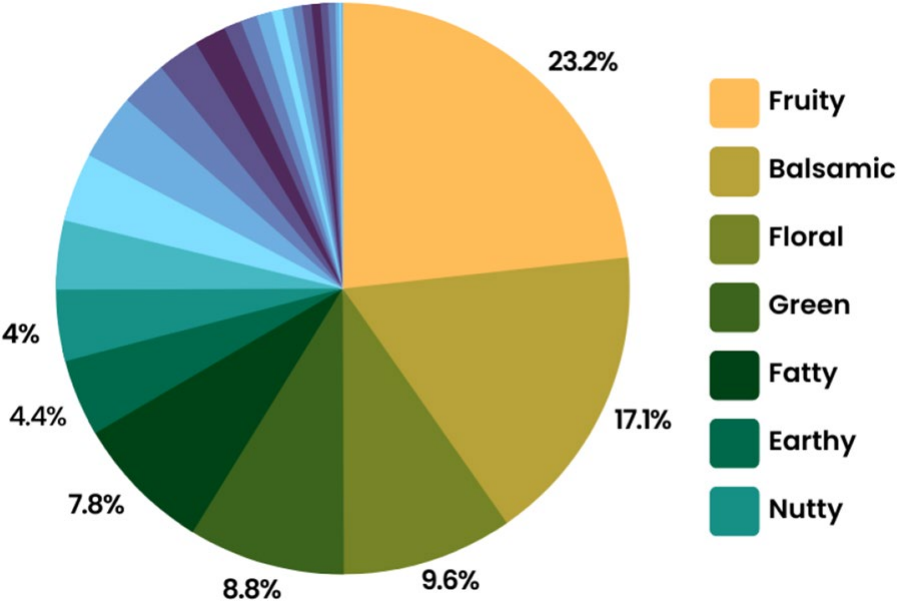
The seven most frequent flavor categories identified in FooDB

Herein, we used food chemicals to generate visual representations of the chemical space as artworks. Food and its flavors, colors, textures, and aromas are generally associated with the great pleasures of life; for this reason, they have been a source of inspiration in art world. However, an approximation at the structural level of the molecules has yet to be addressed. Specifically, we used chemical structures from the public database FooDB [25]. The current version of FooDB contains 70,477 compounds, and after data set standardization (described in detail in Sect. "Data set standardization") has 52,856 molecules. FooDB has information about macronutrients, micronutrients, and food chemicals that give food flavor, color, taste, texture, and aroma to foods. Each chemical item in FooDB contains more than 100 separate data fields providing detailed compositional, biochemical, and physiological information [25]. From FooDB, 4964 natural flavorings derived from food compounds were identified across twenty flavor categories. Figure 2 summarizes the frequency of the seven most populated categories.

From the twenty-seven flavor categories, we defined four new flavor categories: (1) ground flavors, (2) wine-tasting, (3) contrast between fatty and spicy, and (4) natural remedies. Additional file 1: Table S1 shows the number of compounds in each of the four categories considered in this work. Flavors of the ground/flavor similar to herbaceous are earthy, herbaceous, and green flavors. Wine tasting is composed of fruity and floral flavors. The contrast between fatty and spicy is composed of fatty and spicy flavors. Medicinal comprises balsamic, chemical, and medicinal, which are characteristic flavors found in ointments, alcohol, and syrups. Additional file 1: Fig. S1 shows the overlapping compounds between the selected flavor categories.

### Data set standardization

Compounds in FooDB, encoded as SMILES strings [12], were standardized using the open-source cheminformatics toolkit RDKit [26] and Standardizer, LargestFragmentChoser, Uncharger, Reionizer y TautomerCanonicalizer functions implemented in MolVS [27]. Compounds with valence errors or any chemical element other than H, B, C, N, O, F, Si, P, S, Cl, Se, Br, and I were removed. Stereochemistry information, when available, was retained. Compounds with multiple components were split, and the largest component was retained. The remaining compounds were neutralized and reionized to generate the corresponding canonical tautomer.

### Molecular descriptors

For each molecule, physicochemical properties and molecular fingerprints were calculated as descriptors using Python language and RDKit. The whole molecule descriptors computed were hydrogen bond donors (HBD), hydrogen bond acceptors (HBA), topological polar surface area (TPSA), number of rotatable bonds (RB), molecular weight (MW), and partition coefficient octanol/water (LogP). Molecular fingerprints computed were Molecular Access System (MACCS) Keys (166-bits) [13], extended connectivity fingerprint (ECFP) [14] of 1024-bits with diameter 4 (ECFP4). Of note, virtually any other descriptors can be used, as further commented in the Sect. "Discussion".

### Visualization methods

In this study, we used three well-known dimensionality reduction methods: t-SNE, PCA, and TMAPs, although additional visualization methods can be used. Briefly, t-SNE generates plots that organize compounds. Similar compounds form clusters and dissimilar compounds are distant from each other. PCA is a linear dimensionality reduction technique that transforms data with many dimensions (i.e., descriptors) into a lower dimensional space and keeps the different relationships between the data points as much as possible [18]. PCA was generated from six whole molecule descriptors (MW, HB, HBA, SlogP, TPSA, and RB). TMAPs allow visualization of many chemical compounds through the distance between clusters and the detailed structure of these through branches and sub-branches. Local sensitive hashing allows each compound to be grouped hierarchically according to common substructures using molecular fingerprints. In this work, we use MACCS keys (166-bits) [13] fingerprints. Then, each chemical compound was encoded using the MinHash algorithm. The number of nearest neighbors, k = 50, and the factor used by the augmented query algorithm, kc = 10, were used to generate the TMAPs [20].

## Results

**Fig. 3:**
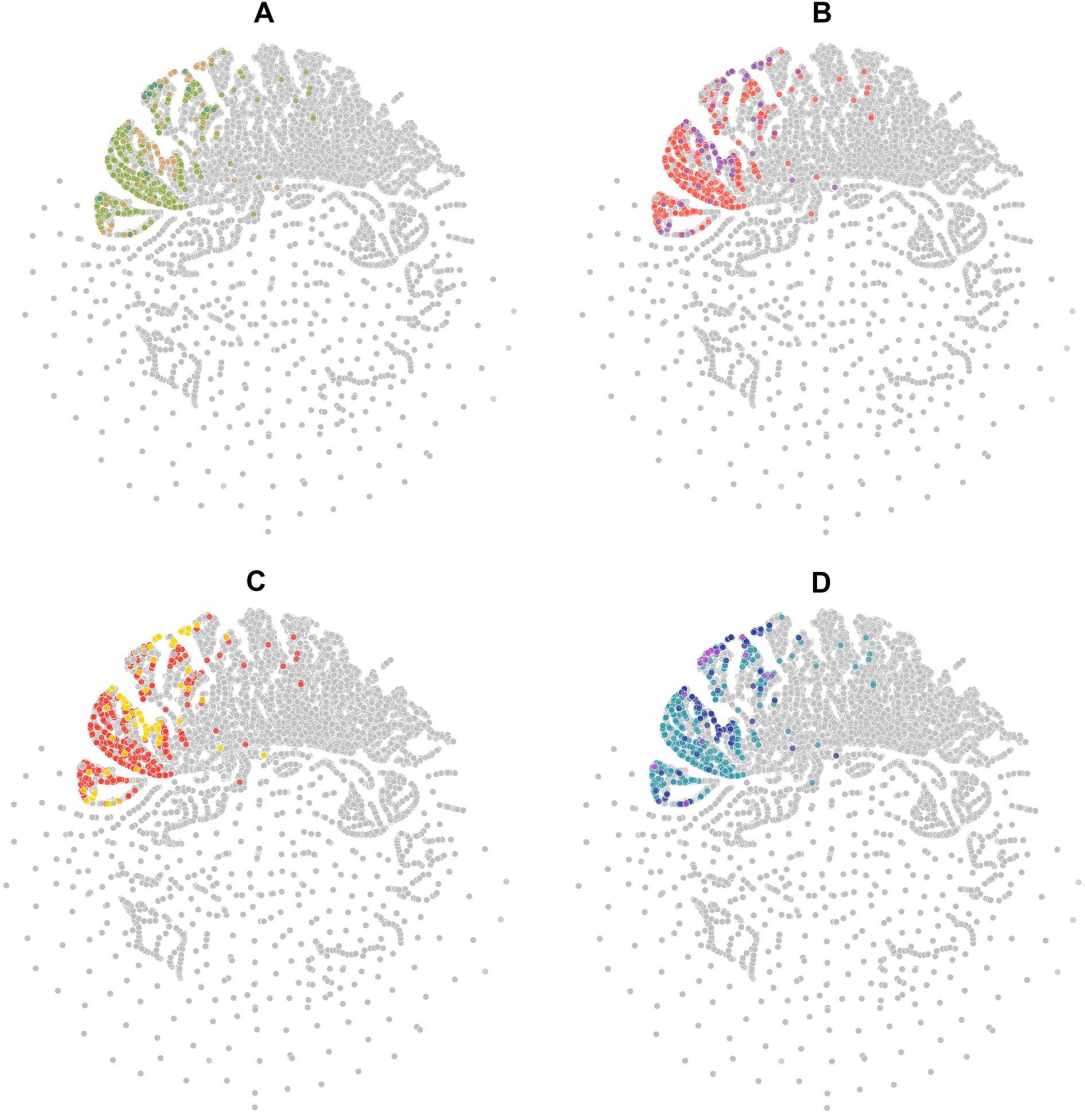
Four flavor categories and full FooDB. The flavor categories are **A** Ground flavors (655 compounds), **B** Wine-tasting (1024 compounds), **C** Contrast between fatty and spicy (430 compounds), and **D** Natural remedies (762 compounds)

**Fig. 4:**
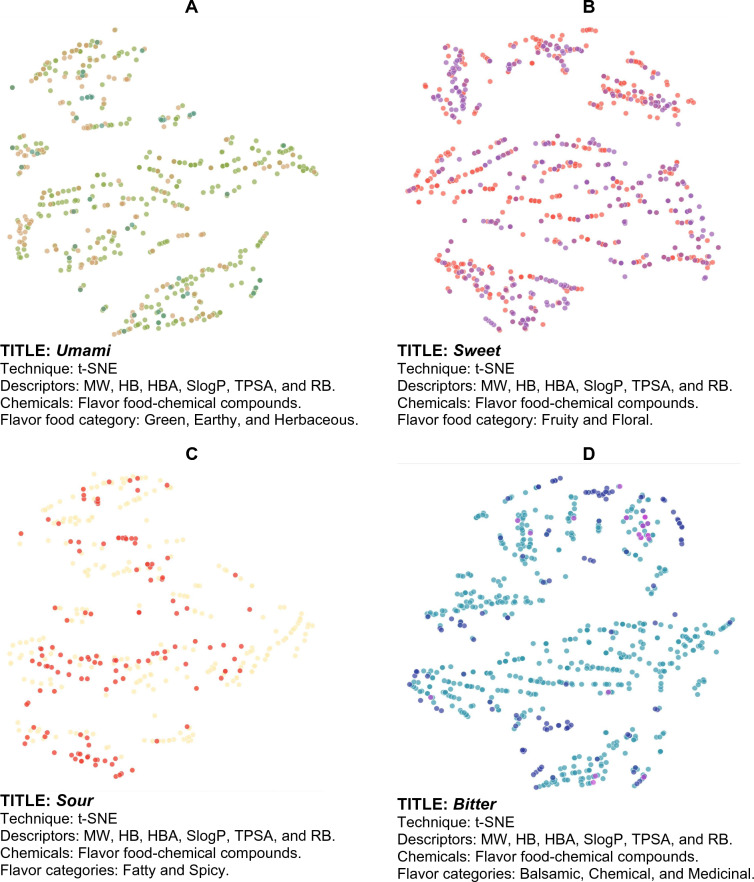
Four flavor categories: **A** Ground flavors (655 compounds), **B** Wine-tasting (1024 compounds), **C** Contrast between fatty and spicy (430 compounds), and **D** Natural remedies (762 compounds)

**Fig. 5:**
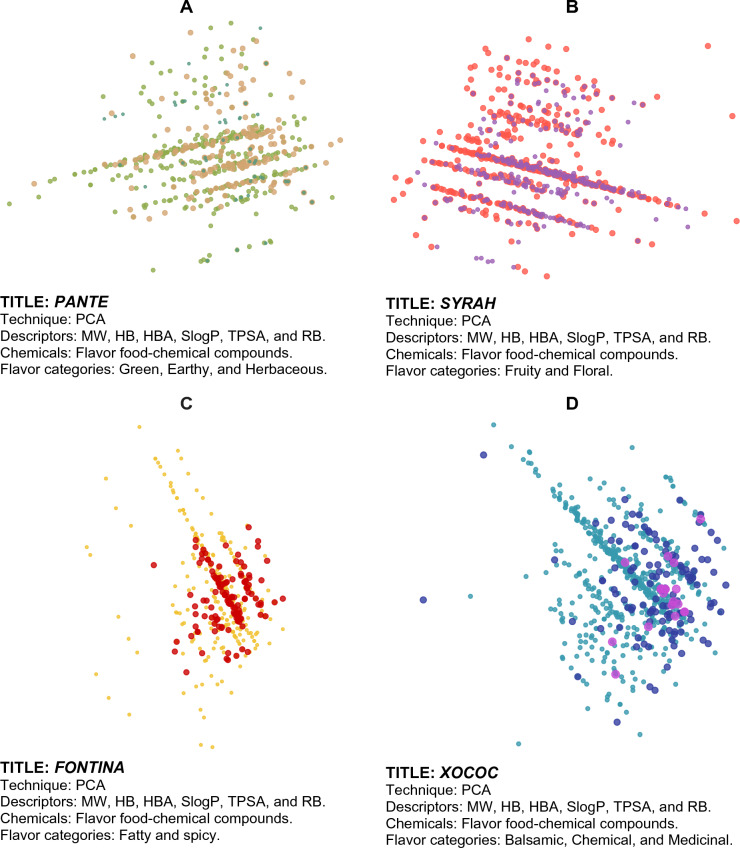
Four flavor categories: **A** Ground flavors (655 compounds), **B** Wine-tasting (1024 compounds), **C** Contrast between fatty and spicy (430 compounds), and **D** Natural remedies (762 compounds)

**Fig. 6:**
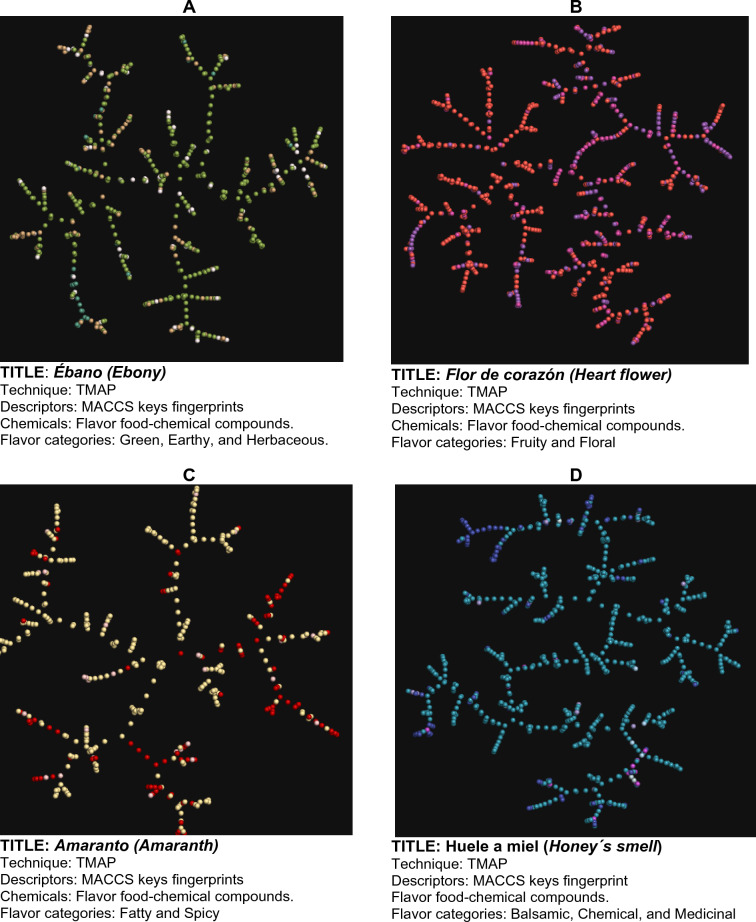
Four flavor categories: **A** Ground flavors (655 compounds), **B** wine-tasting (1024 compounds), **C** Contrast between fatty and spicy (430 compounds), and **D** Natural remedies (762 compounds)

Figures 3, 4, 5, 6 show examples of so-called “Art Galleries” composed by visualization of the chemical space of different food chemical categories. The visual representations of chemical space were generated with t-SNE (Figs. 3 and 4), PCA (Fig. 5), and TMAPs (Fig. 6). Below each image (i.e., “digital paintings”) is presented basic information of the “technique” (visualization method, allusive to the techniques used in paintings), descriptors, and chemicals (that would be meaningful information for a chemistry-oriented person to understand the data presented). Each visual representation of the chemical space or Artwork includes a “Title” that is reminiscent of the name of the piece of art or digital painting.

## Discussion

Chemoinformatics has been broadly used in drug discovery. Still, it has many more applications in chemistry, with increasing applications in food chemistry, as evidenced by the emergence of the research areas of food chemical informatics or food informatics [28, 29]. There are others, such as natural products [30, 31], polymers, and materials, to name a few [6]. Herein, we propose expanding the realm of chemoinformatics´ applications through the visual representation of the chemical space of compound data sets—herein illustrated with food chemicals—to yield exemplary “art pieces.” The connection or synergy between chemoinformatics and art has a strong potential to bring together at least two sectors of the population that might be otherwise disconnected. From an educational point of view, which is a central need in chemoinformatics—the synergy might attract young students and kids to chemistry through art.

The subdiscipline of food informatics was proposed in 2014 as a specific application of chemoinformatics to food chemistry [28]. Since then, numerous applications of chemoinformatics to different aspects of food chemistry have been published, including analysis of the chemical space of food chemicals to characterize the structural diversity [32]. In Sect. "Results" we showed examples of visual representations of the chemical space of food chemicals as an artistic expression and scientific dissemination through art. There are many possibilities to expand the genesis of the proposed “art-cheminformatics,” as further elaborated in Sect. “Conclusions and outlook”.

### Exemplary art-related chemical spaces and multiverses

The examples of visual representation of chemical space as artistic representations presented in Sect. "Results" are focused on food chemicals and molecular descriptors suitable to represent such chemical compounds. Also, examples of visualization methods used in the previous section are t-SNE, PCA, and TMAPs. However, as commented in the Introduction, the number of established visualization techniques, molecular descriptors, and, perhaps most importantly, the number of chemical structures are immense. Therefore, there are thousands or millions of ways to generate chemical space-driven works of art. To glimpse the artistic possibilities, Table 1 summarizes examples of the cheminformatics-driven visualization of chemical space and multiverses. The table summarizes examples of compound data sets with chemicals of different types that could be used to represent their vastness, complexity, diversity, and chaotic intrinsic features from an artistic perspective. Many more compound data sets and multiple combinations of descriptors and visualization techniques could be used. However, as with any other artistic vehicle, the real importance of any type of art is its capacity to tell histories or convey a message that sometimes is hidden.

**Table 1:** Exemplary potential paintings based on the visualization of the chemical space of compound data sets

Data set	Artistic meaning	Artwork name
Random compounds	Aleatory molecules represent the vastness of our universe and daily life. We are in contact with many chemicals every time, but we don’t look at their complexity and intrinsic disorder in our universe and daily life	“Chaos”
Diverse data set	The diversity offers many colors, flavors, tastes, and experiences. In nature, diversity (in all senses) is a constant feature	“Diversity”
Marine natural products	We don't understand the sea; It has life, death, color, and darkness. It's constantly changing	“The Ocean” “Immensity”
Drugs approved for the treatment of HIV	Everything happens in a positive HIV human; Fear, memories, happiness, and normality. The drugs help… but are not a complete answer	“Living with AIDS”
Hormones—neurotransmitters	Love = hormones + neurotransmitters + special persons	“The chemistry of love”
Chemicals associated with depression	Depression = hormones + neurotransmitters—purpose	“Darkness”
Food chemicals	The great pleasures of life are often accompanied by flavors, colors, textures, and aromas	“Bellyful” “Flavor trip”
ZINC database vs. drug-like compounds	We know a lot about our nature and composition, but we don't know much more. Our knowledge is a mere stain on an entire canvas that we do not yet understand	“Our knowledge”

**Fig. 7:**
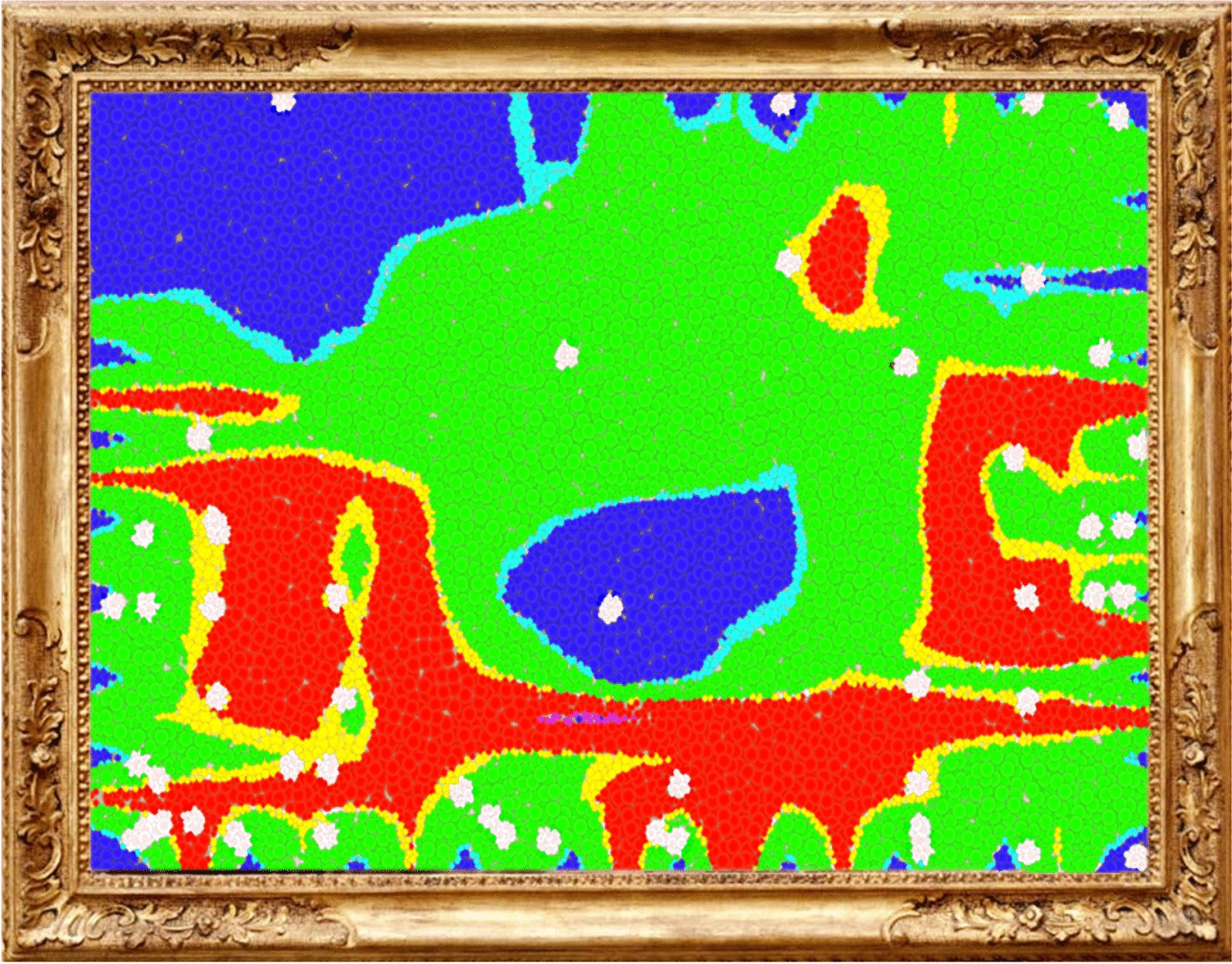
Chemical space art example. Title: “Wise nature”; Autor: Edgar López-López; Technique: SOM—using DataWarrior software [33, 34]; Dataset: Random natural products (1000 compounds); Descriptors: predicted mutagenic, tumorogenic, Reproductive effective, and Irritant; Technical description: Each white point is a natural product, the regions colored in red represent the chemical space with a high predicted probability of containing compounds witch side effects, the opposite for the blue color; Artistic interpretation: The "nature" is not always healthy, in nature, there has always been a duality between what fills us with life and what takes it away

To illustrate further the potential of generating artistic representations through visualization of chemical space, Fig. 7 shows an example of chemical space artwork from a random natural products dataset, decoding by their side effects descriptors (e.g., mutagenesis, tumorogenesis, and negative reproductive effects, etc.). Their color palette, from red to blue, represents the probability of each natural product generating side effects. The “canvas” was “painted” with a dotted technique, reflecting another possible set of textures that can be developed with this technique. Like in Fig. 7, we intrinsically know that "nature" is not always healthy and that within us, there is a delicate balance that is very easy to break.

**Fig. 8:**
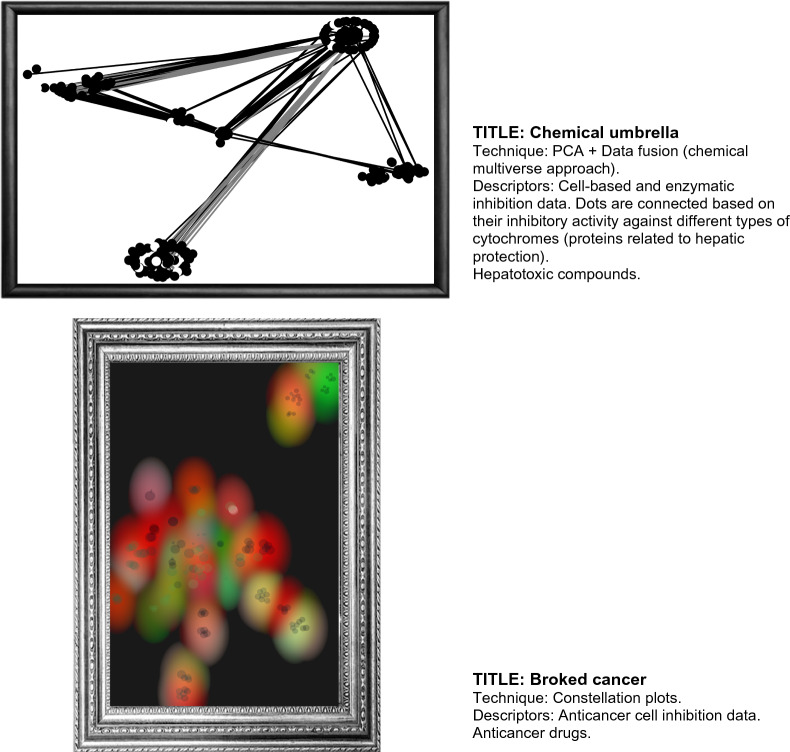
Chemical space art examples. Chemical artworks were generated with public data [35–37]

Figure 8 shows additional examples of chemical space artwork that combine different reduction data methods and descriptors to generate an artistic visual representation of the chemical data. We encourage the readers to reflect and find other artistic interpretations that these figures could have. The examples of chemical space visualization as work art have been included in a Chemical Space Art Gallery freely available at https://www.difacquim.com/chemical-art-gallery/

### Artificial intelligence and digital art

**Fig. 9:**
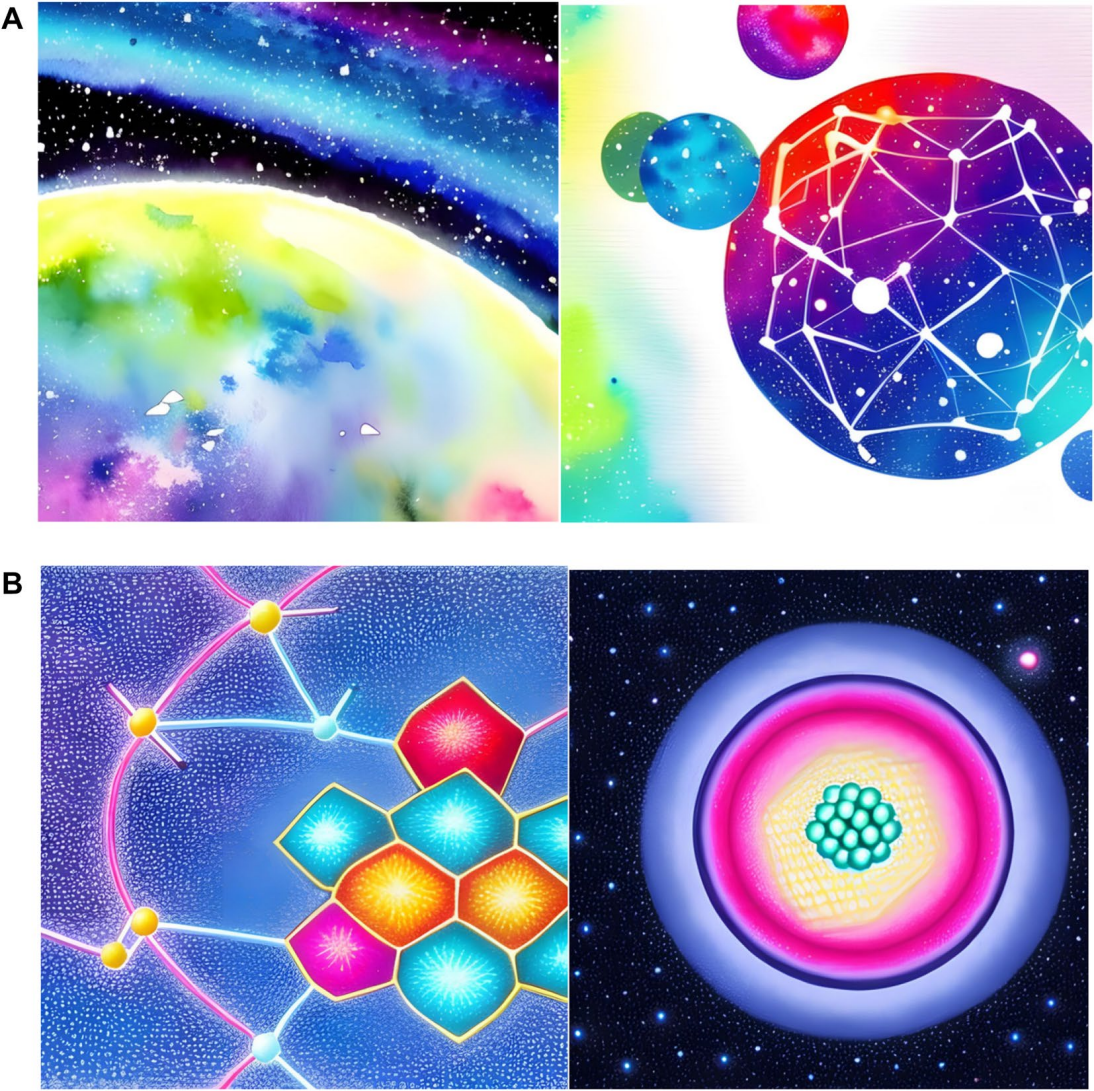
Example(s) of artificial intelligence-driven art with the free application Canva (https://www.canva.com/) using the keyword chemical space and **A** Watercolor and **B** color pencil

Artificial intelligence (AI) is used to generate artistic representations [38, 39]. Although it is not the central point of this manuscript, Fig. 9 illustrates images generated with free resources using keywords associated with “chemical space.” Specifically, the figure shows an example of a chemical multiverse/chemical space driven by an AI-web server training on words. Although the images are attractive, a striking difference with the chemical space artworks presented in previous sections (Figs. 3, 4, 5, 6, 7, 8) is that the images in Fig. 9 are based on keyword training. The former are derived directly from chemical structures encoded with molecular descriptors. Another important aspect is a greater understanding and human intervention in the former representations, something questionable in AI-guided pictures.

## Conclusions and outlook

Science and art have long been intimately related. A typical example is summarized by the phrase, “Drug discovery is as much an art as it is a science.” Certainly, chemistry is substantially used in art, such as in art restoration and preservation. However, an emerging trend exists to apply chemistry and its concepts to generate artwork. Herein, we discuss an approach to combining art with chemoinformatics through the visual representations of chemical space. We presented a few examples of chemical space artworks that can be “digital paintings.” The author of the low-dimensional graphs can use the plots with dual general purposes: communicate data and generate chemical information (as generally done with the visualizations of chemical space) and convey an emotional or personal meaning to the graph (driven by chemistry and informatics principles).

We also conclude that chemical space-driven works of art can be tools to promote science in general and chemistry in particular for the broad audience. Thus, chemistry informatic-driven artistic expressions can be an approach to disseminating science. Such an approach aligns with the graphical abstracts frequently used in peer-reviewed journals. The "chemical art" could be useful to represent complex data by using an artistic and attractive perspective. The person generating the chemical space representation could be considered a “chemical space artist.”

We envision several further developments and areas of opportunity for art driven by visual representations of chemical space. Table 2 summarizes ongoing chemical arts projects, from the generation of “easy to use” tools, the first chemical art gallery, and the implementation of this artistic mode to introduce the new generation of chemoinformaticians to the chemical space concept. In parallel, AI methods will continue expanding and exploring the chemical space, offering new types of molecules and descriptors that could be used to increase the possibilities of representing chemical space from an artist's perspective.

**Table 2:** Representative developments of combining art with chemoinformatics through artistic visualizations of chemical space

Development	Putative outcome or application
Continue developing a digital collection focused on the artistic representation of the chemical space	*The Chemical Space Art Museum*
Generate automated workflows using open software or informatic tools to improve the accessibility of this kind of art to people with different academic/artistic backgrounds	*ChemArt Generators*
Establish a free, open-access, and permanent repository of art pieces. This encourages open science and open art. The scientific and artistic community could support the repository	*ChemART Gallery.* An example is at https://www.difacquim.com/chemical-art-gallery/
Set up a sustained educational or cultural program as a continued open and permanent exposition	*Art Driven by Chemical Space Visualization* program

### Supplementary Information

**Additional file 1: Table S1.** The number of flavor compounds, flavor notes, and flavor categories. **Figure S1.** Unique and overlapping structures of four flavor categories from FooDB. All the code and data sets to reproduce the visual representation of the chemical space presented in the manuscript are freely available at https://github.com/DIFACQUIM/Art-Driven-by-Visual-Representations-of-Chemical-Space-.

## Data Availability

All data related to this manuscript can be accessed in the Supplementary material.

## Abbreviations

AI: Artificial intelligence
ECPF: Extended connectivity fingerprint
HBD: Hydrogen bond donors
HBA: Hydrogen bond acceptors
GTM: Generative topographic mapping
LogP: Partition coefficient octanol/water
MACCS: Molecular ACCes System
MW: Molecular weight
PCA: Principal component analysis
TMAP: TreeMap
t-SNE: T-Distributed stochastic neighbor embedding
TPSA: Topological polar surface area
SOM: Self-organizing map
