# Supplementary material for: Art driven by visual representations of chemical space
Source: J Cheminform. 2023 Oct 21;15:100. doi: 10.1186/s13321-023-00770-4 (PMC10590523; doi:10.1186/s13321-023-00770-4)
Supplement: Supplementary file 1 — Additional file 1: Table S1. The number of flavor compounds, flavor notes, and flavor categories. Figure S1. Unique and overlapping structures of four flavor categories from FooDB. All the code and data sets to reproduce the visual representation of the chemical space presented in the manuscript are freely available at https://github.com/DIFACQUIM/Art-Driven-by-Visual-Representations-of-Chemical-Space-. [file 13321_2023_770_MOESM1_ESM.docx]

**ADDITIONAL MATERIAL**

**Art Driven by Visual Representations of Chemical Space**

Daniela Gaytán-Hernández,^1^ Ana L. Chávez-Hernández,^1^ Edgar López-López,^1,2^ Jazmín Miranda-Salas,^1^ Fernanda I. Saldívar-González,^1^ José L. Medina-Franco*^,1^

*^1^ DIFACQUIM Research Group, Department of Pharmacy, School of Chemistry, Universidad Nacional Autónoma de México, Avenida Universidad 3000, México City 04510, Mexico*

*^2^ Department of Chemistry and Graduate Program in Pharmacology, Center for Research and Advanced Studies of the National Polytechnic Institute, Mexico City 07000, Mexico*

**Contents**

|  |  | Page |
| --- | --- | --- |
| **Table S1** | The number of flavor compounds, flavor category, and exemplary flavors. | S2 |
| **Figure S1** | Unique and overlapping structures of four flavor categories from FooDB. | S3 |

**Table S1.** The number of flavor compounds, flavor category, and exemplary flavors. A) Ground’s flavors, B) Wine-tasting, C) Contrast between fatty and spicy, and D) Natural remedies.

| Flavor notes | Flavor category | Exemplary flavors | Number of compounds |
| --- | --- | --- | --- |
| Earthy | Ground’s flavors | Mushrooms (Earthy)  Wintergreen (Green)  Clove (Herbaceous) | 655 |
| Green |  |  |  |
| Herbaceous |  |  |  |
| Fruity | Wine-tasting | Banana, pineapple (Fruity)  Lavender, Rose (Floral) | 1024 |
| Floral |  |  |  |
| Fatty | Contrast between fatty and spicy | Cheese, butter (Fatty)  Coumarin (Spicy) | 430 |
| Spicy |  |  |  |
| Balsamic | Natural remedies | Vanilla, Cinnamon (Balsamic)  Alcohol (Chemical)  Chavicol (Medicinal) | 762 |
| Chemical |  |  |  |
| Medicinal |  |  |  |

| **A**  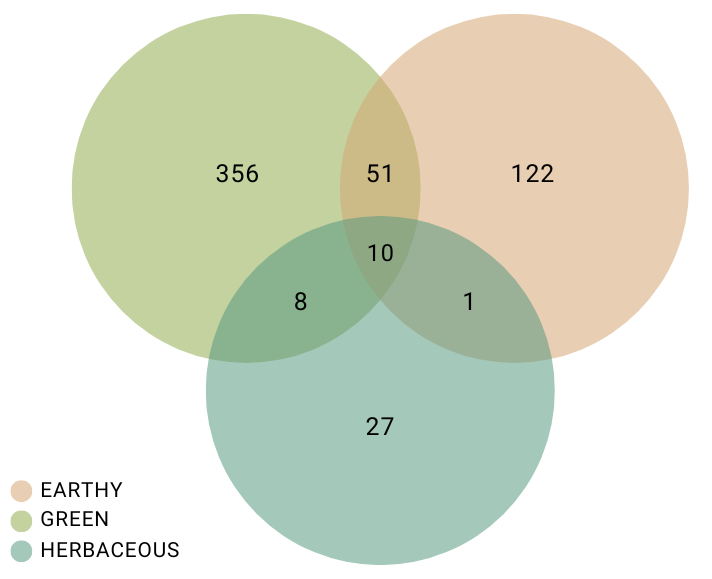 | **B**  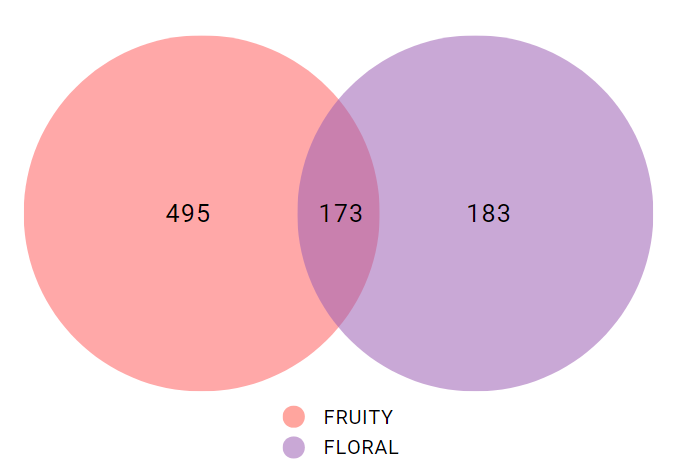 |
| --- | --- |
| **C**  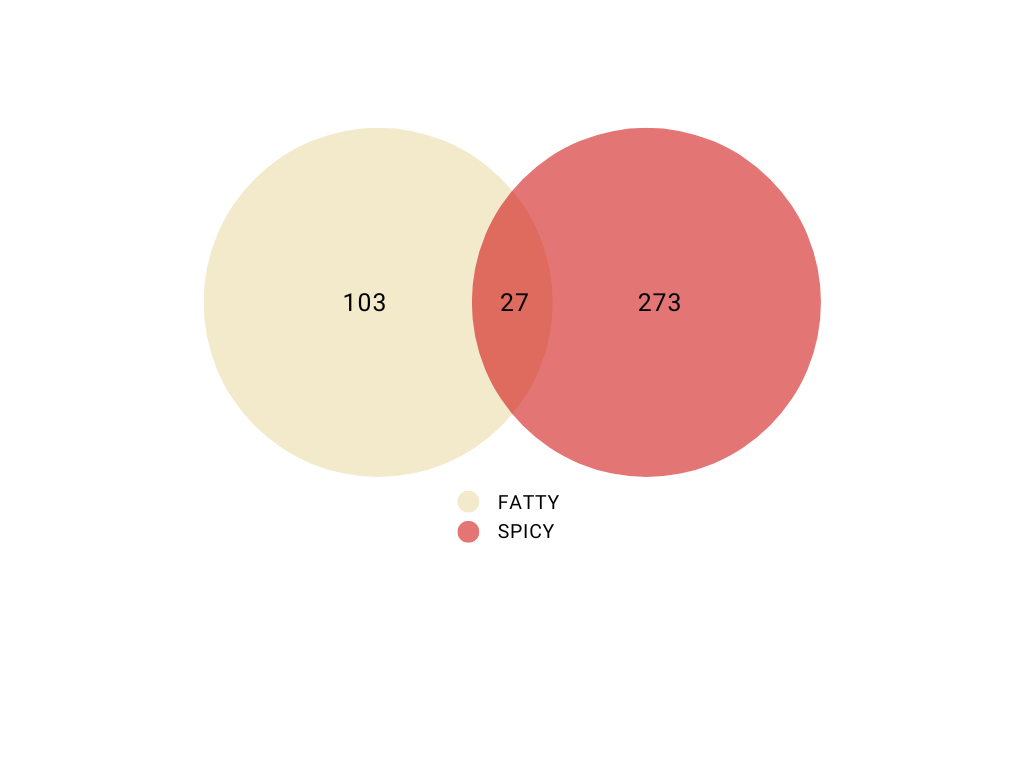 | **D**  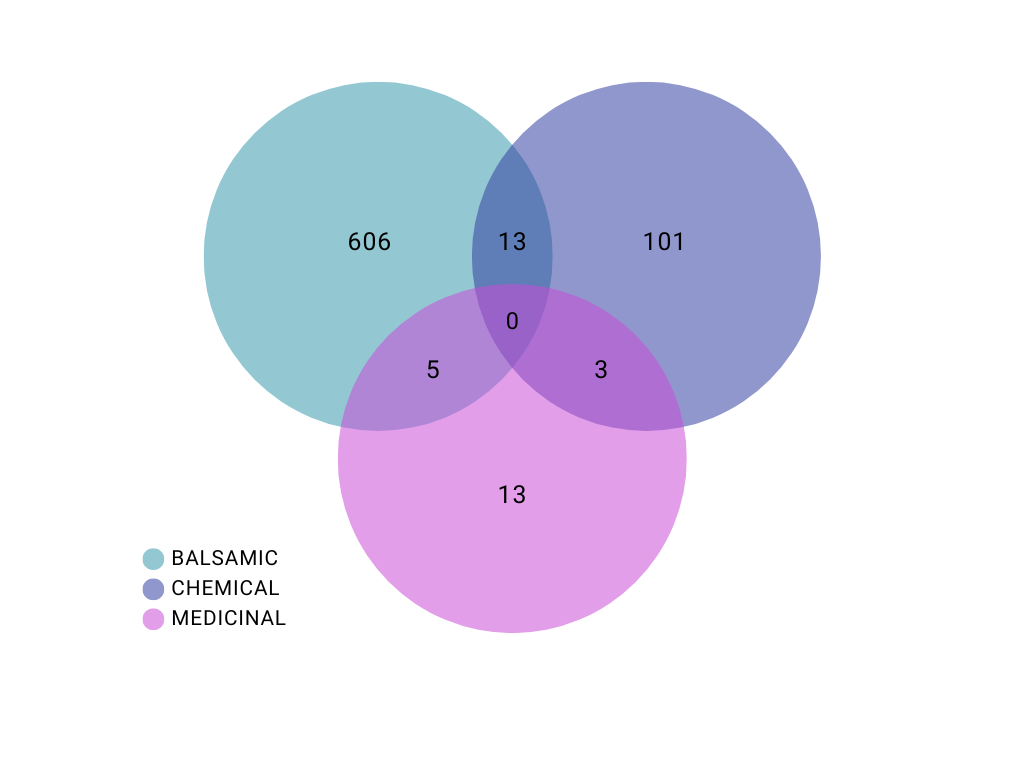 |

**Figure S1.** Unique and overlapping structures of four flavor categories from FooDB. A) Ground’s flavors B) Wine-tasting, C) Contrast between fatty and spicy, and D) Natural remedies.
